# An Active Contour Model Based on Adaptive Threshold for Extraction of Cerebral Vascular Structures

**DOI:** 10.1155/2016/6472397

**Published:** 2016-08-15

**Authors:** Jiaxin Wang, Shifeng Zhao, Zifeng Liu, Yun Tian, Fuqing Duan, Yutong Pan

**Affiliations:** ^1^College of Information Science and Technology, Beijing Normal University, Beijing 100875, China; ^2^Beijing Key Laboratory of Digital Preservation and Virtual Reality for Cultural Heritage, Beijing 100875, China

## Abstract

Cerebral vessel segmentation is essential and helpful for the clinical diagnosis and the related research. However, automatic segmentation of brain vessels remains challenging because of the variable vessel shape and high complex of vessel geometry. This study proposes a new active contour model (ACM) implemented by the level-set method for segmenting vessels from TOF-MRA data. The energy function of the new model, combining both region intensity and boundary information, is composed of two region terms, one boundary term and one penalty term. The global threshold representing the lower gray boundary of the target object by maximum intensity projection (MIP) is defined in the first-region term, and it is used to guide the segmentation of the thick vessels. In the second term, a dynamic intensity threshold is employed to extract the tiny vessels. The boundary term is used to drive the contours to evolve towards the boundaries with high gradients. The penalty term is used to avoid reinitialization of the level-set function. Experimental results on 10 clinical brain data sets demonstrate that our method is not only able to achieve better Dice Similarity Coefficient than the global threshold based method and localized hybrid level-set method but also able to extract whole cerebral vessel trees, including the thin vessels.

## 1. Introduction

Cerebral vascular diseases have become the main incentives to dizziness, disability, and even death in many countries around the world, and the research for vessels arouses concern. The segmentation of cerebral vascular structures is important for the clinical diagnosis and analysis. In medical image processing field, segmentation means the extraction of anatomical structures of interest from original data [1, 2]. Because of low contrast of images, edge blur, and structure complexity of cerebral vessels, the accurate segmentation is still a challenging task and deserves to be researched [3, 4].

Over the past few decades, a large number of methods for vessel segmentation have been proposed, including atlas-based techniques [5–10], machine learning techniques [11–14], and active contour model (ACM) [15, 16]. A comprehensive review can be referred to in Lesage et al. work [17]. Among these techniques, the ACM has been widely applied in medical image segmentation because of its easy extensibility. The ACM is based on geometric curve evolution theory and the essential idea of that technique is to evolve the initial curve or surface to the boundaries of target objects driven by internal forces and external forces [18]. Active contours can be implicitly presented by the level-set methods, which put original curves into higher dimensional spaces to research and are achieved in numerical computations by the Eulerian approach [19].

ACMs using level-set formulation have various forms of expression, and they are divided into three major categories: edge-based, region-based, and hybrid level-set models. In edge-based models, edges are usually generated first by an edge-detection algorithm and then using postprocessing to adjust to the final boundaries [20]. The typical edge-based model is the geodesic active contour model [21]. The model combines active contours with the computation of geodesic distance curve, and it allows to associate classical snakes based on energy minimization with geometric active contours based on the theory of curve evolution.

A method using a new indicator (i.e., salient edge energy) to guide a given contour robustly and accurately towards the target object boundary was proposed by W. Kim and C. Kim [22]. They defined the salient edge energy by exploiting the higher order statistics on the diffusion space and embedded it into a variational level-set function. But the edge-based models are sensitive to noise and seek to oversegment an image.

Region-based models are built on using the similarity among pixels to form homogeneous regions in an image [20]. The Mumford-Shah (M-S) model is the typical technique of that, and it depends on the defined edge function based on image gradient to stop the evolution process of active contour curves. When the contour curve is closer to the boundary of a target object, the value of gradient is higher, which causes the edge function to be closer to zero, and the evolutionary curve stops at the location of boundaries [23].

Based on the M-S model, Chan and Vese proposed the famous C-V model [24]. The C-V model can detect objects whose boundaries are not necessarily defined by gradient, because the stopping term defined in the energy function depends on the gradient of an image and is instead associated with particular segmentation of the image. In addition, the authors give a numerical algorithm using finite differences. Based on the C-V model, Tian et al. [25] proposed embedding local intensity weighting and a vessel vector field into the vessel active contour model. However, the model needed to be improved to better match segment 3D vessels. In these methods, it is essential to reinitialize the level-set function to make it close to the signed distance function [26]. However, the periodic reinitialization is time-consuming, and it is difficult to prevent the level-set function from being too steep or flat during the evolution [27]. To solve the problem, Li et al. [28] proposed a method through embedding the penalty term for penalizing the deviation of the level-set function from a signed distance function into the energy function.

Combining region-based methods with other information, Said [29] proposed a robust level-set-based multiregion and texture image segmentation approach. Zhang et al. [30] proposed a method to associate interactively specified regions of interest with the active contour model while keeping the user interaction to the minimum. Sciolla et al. [31] proposed a multigrid level-set segmentation method based on a region-based function, the Hellinger distance. Jiang et al. [32] used the hybrid level-set method with a nonlinear speed function to extract brain from cerebral MRI volume. Zhao et al. [33] developed a MIP-guided approach for brain vessel segmentation. They first projected the volume onto the 2D plane, applied an integrated active contour model to extract blood vessels from MIP images, and then projected back to the 3D volume. The proposed method showed satisfying segmenting results. However, their method is a little complicated with several projection and back projection operations.

In this study, we propose an ACM implemented by the level-set method in order to segment cerebral vascular structures from TOF-MRA data. We consider both the region information and edge information and combine them to characterize the energy function. A fixed gray threshold is used into the region term to represent the global information. In addition, we embed the adaptively dynamic threshold into our model to depict local region information, which is helpful for segment more integrated vessels. To avoid reinitializing level-set function in every evolution, the penalty term proposed by Li et al. is extended to 3D and applied into our model.

The organization of this study is as follows. In Section 2, we will introduce the related works. In Section 3, the proposed segmentation methodology is depicted. Experimental validations and discussion are given in Section 4.  Section 5 concludes the paper.

## 2. Related Works

Considering both region information and boundary information and combining them to characterize the energy function is a good idea for segmenting the complex objects. Zhang et al. [34] proposed a hybrid level-set (i.e., HLS) method for segmentation of medical images. They use preset *μ* value which represents the lower gray-level of target object to replace *μ*_in_ and *μ*_out_ in the region term of traditional C-V model, and the geodesic active contour model is applied to represent the edge term. The definition of energy function to be minimized is defined as

(1) $$\begin{array}{c} \varepsilon \left(\;\phi \;\right)=-\alpha \overset{}{\underset{\Omega }{\int }}\left(\;I-\mu \;\right)H\left(\;\phi \;\right)d\Omega +\beta \overset{}{\underset{\Omega }{\int }}g\left|\;\nabla H\left(\;\phi \;\right)\;\right|d\Omega , \end{array}$$

where *I* is the image to be segmented, *Ω* is the image domain, *α* and *β* are weighting factors to balance the first-region term and the second-boundary term, and the zero level set of level-set function *ϕ* represents the active contour. *H*(*ϕ*) is the Heaviside function defined as

(2) $$\begin{array}{c} H\left(\;\phi \;\right)=\left\{\begin{array}{cc} 1, & \text{if }\phi \geq 0\\ 0, & \text{if }\phi <0. \end{array}\right. \end{array}$$

Parameter *μ* is preseted representing the lower gray boundary of the object to be segmented, which means it will extract the object with gray higher than *μ*. However, since the value of *μ* is fixed, it cannot fit the wide intensity distribution of vessels well, especially for those small thin ones.

To solve the problem, Hong et al. [35] proposed a localized hybrid level-set (i.e., LLS) method for the segmentation of 3D vessel images, and they calculated locally specified dynamic threshold *μ*(**u**) to indicate the lower bound of target object and embedded the local gray information into the region term. Defined function *μ*(**u**) is

(3) $$\begin{array}{c} \mu \left(\;u\;\right)=\frac{k*K_{\sigma }\left(u\right)*\left[H\left(\phi \left(u\right)\right)I\left(u\right)\right]}{K_{\sigma }\left(u\right)*H\left(\phi \left(u\right)\right)}, \end{array}$$

where **u** ∈ *Ω*, *k* ∈ [0.5,1] is an adjusting coefficient for preventing the active contours stopping evolution inside the target areas before reaching the boundary, and *K*_*σ*_ is the Gaussian kernel function characterizing the intensity profile of a blood vessel cross section, such as

(4) $$\begin{array}{c} K_{\sigma }\left(\;u\;\right)=\frac{1}{2\pi \sigma^{2}}{e^{-}}^{{\left|u\right|}^{2}/2\sigma^{2}} \end{array}$$

which is used to convolve with the image in order to detect the main vessels. By the use of dynamic threshold *μ*(**u**) defined in (3), the method can segment the tiny vessels better, but it may lose some intensity information of thick parts due to dynamic *μ*(**u**) formulation limitations.

In any way, the above two methods both have respective pros and cons. Former preseted *μ* value drives the contours to enclose thick vessel boundaries with gray-levels greater than *μ*, but it can not perform well on tiny vessels. The latter with dynamic *μ*(**u**) value can deal with the tiny vessels better but does not extract the thick vessels completely. Thus, in our study, we take full advantage of these two methods. Meanwhile, we also extend the penalty term proposed by Li et al. [28] from 2D segmentation to 3D application and embed it into the energy function to keep the characteristic of a signed distance function.

## 3. Proposed Methodology

### 3.1. Definition of the Energy Function

To tackle 3D cerebral vessel segmentation, we propose a new hybrid level-set model (i.e., NHLS model) inspired by models in [34, 35]. To segment more integral vessels, we incorporate dynamic *μ*(**u**) value to the original hybrid model, and the proposed energy function is defined as follows:

(5) εϕ=−α1∫ΩI−μ0HϕdΩ−α2∫ΩI−μuHϕdΩ+β∫Ωg∇HϕdΩ+γPϕ,

where *I* is the 3D volume data to be segmented, *Ω* is data domain, **u** ∈ *Ω*, *μ*_0_ is set based on the lower gray-level boundary of the target object, and *μ*(**u**) is calculated according to (3) representing the local threshold.

In (5), the four terms play different roles. The first term represents the global region information which drives the active contour curve to get close to the regions with bigger intensity value than *μ*_0_. The second term is used to represent local region information which adaptively adjust to the threshold to segment the local tiny parts. The role of the third-boundary term is equivalent to the geodesic active contour model, and it encourages the contour curve to enclose the regions with high image gradient. Parameters *α*_1_, *α*_2_ and *β* are used to balance the two region terms and one boundary term. And the fourth term is the penalty term, in which *γ* is a preseted parameter controlling the effect of penalizing the deviation of *ϕ* from a signed distance function, and *P*(*ϕ*) is the penalty term to avoid reinitializing *ϕ* in evolution, which is defined as

(6) $$\begin{array}{c} P\left(\;\phi \;\right)=\overset{}{\underset{\Omega }{\int }}\frac{1}{2}{\left(\;\nabla \phi -1\;\right)}^{2}d\Omega . \end{array}$$

The related PDE can be derived from the gradient decent flow applied to functional (5):

(7) ∂ϕ∂t=δϕ·α1I−μ0+α2I−μu+β div⁡g∇ϕ∇ϕ+γΔϕ−div⁡∇ϕ∇ϕ.

### 3.2. Implementation

Edge function *g*(·) represents the regularized gradient map used for geodesic active contour and nonlinear diffusion related to boundary feature of the image. In this study, *g*(·) is defined as

(8) $$\begin{array}{c} g\left(\;x\;\right)=\frac{1}{\left(1+x^{2}\right)}. \end{array}$$

Function *H*(·) is the Heaviside function and the original function is not continuous; therefore, it cannot fit the smooth boundary curve of the practical object. To solve this problem, it is usual to use a kind of smooth function to replace the original one. There are various proposed smooth types of the Heaviside function. We adopt the smooth Heaviside function *H*(*ϕ*) as follow:

(9) $$\begin{array}{c} H\left(\;\phi \;\right)=\frac{1}{2}\left(\;1+\frac{2}{\pi }\arctan\left(\;\frac{\phi }{\varepsilon }\;\right)\;\right). \end{array}$$

And the definition of corresponding Dirac function *δ*(*ϕ*) is

(10) $$\begin{array}{c} \delta \left(\;\phi \;\right)=\frac{1}{\pi }\frac{\varepsilon }{\varepsilon^{2}+\phi^{2}}. \end{array}$$

We use the above computations *H*(*ϕ*) and *δ*(*ϕ*) to replace original *H*(·) and *δ*(·) in (2) and (7), respectively.

Considering the penalty term in (7), Δ is the Laplacian operator, and

(11) $$\begin{array}{c} \Delta \phi -\mathrm{div}\left(\;\frac{\nabla \phi }{\left|\nabla \phi \right|}\;\right)=\mathrm{div}\left[\;\left(\;1-\frac{1}{\left|\nabla \phi \right|}\;\right)\nabla \phi \;\right] \end{array}$$

has factor 1 − 1/|∇*ϕ*| as diffusion rate. If |∇*ϕ* | > 1, the diffusion rate is positive. If |∇*ϕ* | < 1, the diffusion rate is negative.

Equation (7) can be simply written as

(12) $$\begin{array}{c} \frac{\phi^{k+1}-\phi^{k}}{\Delta t}=\alpha_{1}M_{1}+\alpha_{2}M_{2}+\beta N+\gamma P, \end{array}$$

where *ϕ* ^*k*+1^ and *ϕ* ^*k*^ denote the level-set function *ϕ* in (*k* + 1)th and *k*th iterations, respectively, Δ*t* is the preset time step, *M*_1_ is the global region term, *M*_2_ is the local term, *N* is the edge term, and *P* is the penalty term. It is required to illustrate that *M*_1_ is a fixed value decided by* I* and *μ*_0_ is not related to *ϕ*. *M*_2_, *N*, and *P* can be also expressed as *M*_2_(*ϕ* ^*k*^), *N*(*ϕ* ^*k*^), and *P*(*ϕ* ^*k*^), and they are affected by *ϕ* ^*k*^. Difference equation (12) can be represented as follows:

(13) $$\begin{array}{c} \phi^{k+1}=\phi^{k}+\Delta t\left(\;\alpha_{1}M_{1}+\alpha_{2}M_{2}+\beta N+\gamma P\;\right). \end{array}$$

Iteration from *ϕ* ^*k*^ to *ϕ* ^*k*+1^ includes five steps:

(i) Compute dynamic localized threshold *μ*(**u**) according to (3).
(ii) Compute penalty term* P* in terms of (6).
(iii) Calculate *α*_1_ *M*_1_ + *α*_2_ *M*_2_ + *γP*.
(iv) Update *ϕ* ^*k*^ to *ϕ* ^*k*′^ using *ϕ* ^*k*′^ = *ϕ* ^*k*^ + Δ*t*(*α*_1_ *M*_1_ + *α*_2_ *M*_2_ + *γP*).
(v) Update *ϕ* ^*k*′^ to *ϕ* ^*k*+1^ using *ϕ* ^*k*+1^ = *ϕ* ^*k*′^ + Δ*tβN*, which is achieved by the semiexplicit method. In fact, we can also use the explicit method to get *ϕ* ^*k*+1^ directly, which plays the same role with the explicit method. However, explicit methods have limitations in time steps, and they need to set time steps small to keep methods stable. If we use explicit methods, the time steps maybe set small to make sure that the process of evolution maintains stability, which leads to time-consuming process [36]. Thus, we choose the semiexplicit method.

There is an additional problem to address that is to set the initial curve of level set. In this study, we apply Frangi's vessel enhancement algorithm into the original data and then implement the canny detection to get the fuzzy boundary of vessel. That boundary curve is used as the initial curve. By this method, it can make sure that every evolution is around the vessel region, which improves efficiency.

### 3.3. Outlier Removement

Since the intensity value of some nonvessel points are very close to those of vessel points, some nonvessel points (i.e., outlier) exist in the segmentation results. In order to remove the outlier points around vessels as much as possible, we need to consider the shape feature of vessels.

Eigenvalues of the Hessian matrix have been successfully used in blood vessel enhancement [37]. For a 3D volume, we assume that the eigenvalues of the Hessian matrix are sorted as |*λ*_1_| ≥ |*λ*_2_| ≥ |*λ*_3_|. The ideal tubular structure in a 3D volume would have

(14) λ3≈0,λ3≪λ2,λ1≈λ2.

Furthermore, in MRA images, the fact is that vessel structures are brighter than the background and the Frangi's vessel enhancement algorithm makes use of all the eigenvalues of Hessian matrix, and it can consider fully the geometric feature that the eigenvalues represent and suppress the impact of irrelevant points on vessels. They define two geometric ratios *R*_*A*_,  *R*_*B*_, and* S*, respectively, as

(15) RB=λ1λ2λ3,RA=λ2λ3,S=λ12+λ22+λ32,

where *R*_*B*_ gets maximum for a blob structure, *R*_*A*_ differentiates plane structures from line structures because in the latter situation it will be zero, and* S* is the measure to distinguish background which will be slow because the eigenvalues are small in the background. On the basis of the three parameters, Frangi et al. define a vesselness function combining those components as follows:
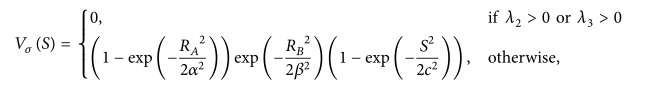
(16)where *α*, *β*, and *c* are thresholds of 3D vesselness function which is used to control the sensitivity of vessel enhancement filter to parameters *R*_*A*_,  *R*_*B*_, and *S*.

As multiscale eigenanalysis of local Hessian operators can enhance local rod-like shapes of varying radii. The value of vesselness function is between 0 and 1. If objects are tubular structures, vesselness function *V*_*σ*_(*S*) is close to 1. For an ideal tubular structure, *R*_*A*_ ≈ 1, *R*_*B*_ ≈ 0. It is noticed that when *R*_*B*_ ≈ 0, the second term in (16) is approximately equal to 1. However, when *R*_*A*_ ≈ 1, the value of the first term in (16) has slight gap with 1. In order to make *V*_*σ*_(*S*) approximately equal 1, we take advantage of function tan⁡(*π*/2)*x*. When *x* ≈ 1, tan⁡(*π*/2)*x* approaches positive infinity, and exp⁡(−tan⁡(*π*/2)(*x*)) is approximately equal to 0, so (1 − exp⁡(−tan⁡(*π*/2)(*x*))) is closer to 1 than before. Thus, in order to enhance the tubular structures to a larger extent, we modified the vesselness function as follows:
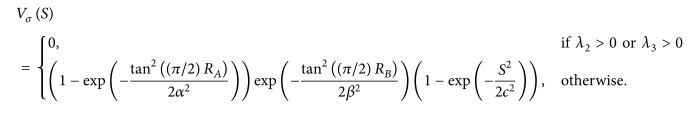
(17)


Considering some available information that can be used to help remove noise may be lost during the process of segmentation; therefore, the algorithm is first used onto the original data to obtain the enhancement vessel structure instead of being applied directly onto the segmentation result. Then, we use the vessel structure to guide the elimination process of nonvessel outlier points in the segmentation result.

## 4. Experimental Results and Discussion

Experiments in extracting cerebral vessels have been conducted on 10 TOF-MRA data sets which were acquired from Navy General Hospital. The 4 sets of data (Data 1, Data 2, Data 3, and Data 4) analyzed in this paper are with the size of 512 × 512 × 216 voxels, the resolution of 0.39 × 0.39 mm^2^, and a slice thickness of 1.2 mm. The experiments are implemented on a computer with Intel® Core*™* i5-4590 CPU 3.30 GHZ CPU, 12.0 G RAM, and Windows 7 operating system. The parameters used are as follows: Δ*t* = 2.0, *α*_1_ = *α*_2_ = 0.003, *β* = 0.02, *γ* = 1.0, *ε* = 1.0.

### 4.1. Comparisons with the HLS and LLS Model

**Figure 1:**
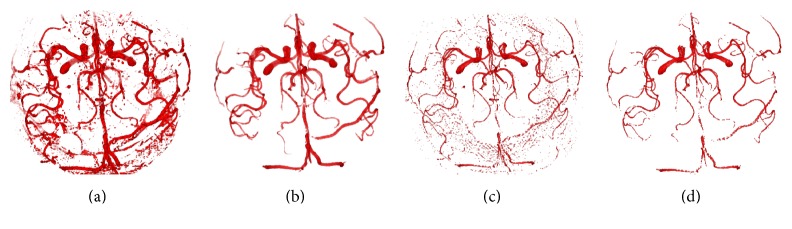
Outlier removement. NHLS segmentation before (a) and after (b) outlier voxels are eliminated with the connectivity filter and LLS segmentation before (c) and after (d) the connectivity filter.

As Figures 1(a) and 1(c) suggest, TOF-MRA is sensitive to fat tissues which would shutter the blood vessels. A circle of points on the top of the head is introduced in the segmented volume due to similar intensity value between tissues and blood vessels. To eliminate them, the volume is processed with an automatic connectivity filter. We first perform the Frangi's vessel enhancement method onto the original MRA data. Then, we preserve the points in our segmentation result that the first step obtained and then start regional growth algorithm using vessel connectivity. Figures 1(b) and 1(d) present the results after applying such a filter to LLS model and our NHLS model.

**Figure 2:**
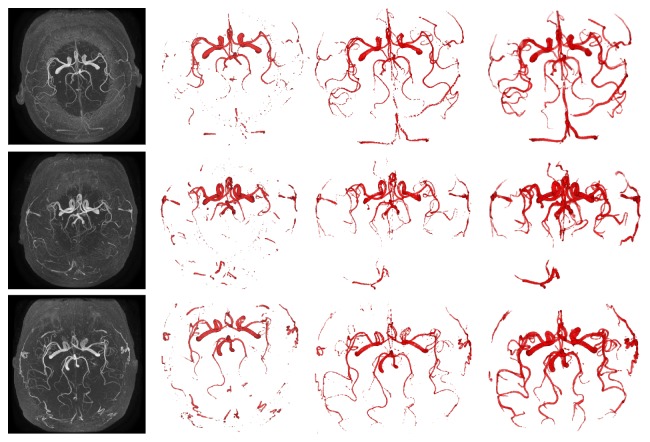
The result of cerebral vascular structures segmented by three models. Each row relates to one patient: the first column represents the MIP images. The second column shows the segmentation result of HLS model; the third column shows the segmentation result of LLS model after noise voxels are eliminated with the connectivity filter; the last column shows the segmentation of NHLS model after noise voxels are eliminated with the connectivity.

All the three methods have been experimented with 10 data sets. Results of the three tests are depicted in Figure 2. The first column of Figure 2 shows the MIP images. The second column shows the segmentation results by HLS model. The third column shows the segmentation results by LLS model. The last column shows the results segmented by our NHLS model.

As for HLS model, through analyzing the histogram of the data set, we notice that the intensity value of cerebral vascular structures is approximately higher than 200. But, there exist differences among different parts of vessels, and for some of them the intensity value may be between 150 and 200. In our experiments, we set low intensity value *μ*_0_ of the three level-set method to be 200; it will extract the vessels with intensity higher than 200 and those lower than 200 will not be extracted as well. The segmentation result is shown in the second column.

As we can see, the segmentation result of cerebral vascular structures is not ideal that it only extracts the large artery structures of vessels but loses many tiny vessel branches. In that method, key parameter *μ*_0_ is predefined to be 200 which means it is unable to extract the small branches with intensity lower than 200. On the other hand, predefined *μ*_0_ is a global threshold; however, the intensity value of different vessel branches is inhomogeneous and has some differences. Therefore, it is essential to consider the local features.

As for LLS model, which is an improved method of HLS model replacing predefined *μ*_0_ with dynamic *μ*(**u**), dynamic *μ*(**u**) is the automatically computed local threshold. The definition of *μ*(**u**) is achieved by the Gaussian kernel function modeling the intensity of a blood vessel cross section. Deviation *σ* of Gaussian kernel is 3.24 in our experiment. The segmentation result is in the third column.

It is noticed that the segmentation result of the second method can extract not only the thick artery structures of vessels but also the tiny branches. On one hand, because the vessel branches are very complex and intensity inhomogeneity occurs in vessel structures, threshold *μ*(**u**) dynamically calculated can characterize the local information of vessels better. On the other hand, using dynamic thresholds representing the lower bound of vessels can consider the regional information better and the segmentation results are more integral than the original one.

However, compared with HLS, the regions of the thick vessels extracted by LLS are not brighter, which means the segmentation result of the thick vessels is not integrated. The defect is caused since the LLS model pays more attention to local and tiny information and neglects some global information, and the segmentation result includes some irrelevant points with the similar intensity value to vessels around the vessels.

The proposed method in this paper is inspired by the HLS model and the LLS model. The result is in the last column. Our model combines the global threshold information with the localized threshold information. We analyze the histogram of data and find that the intensity value of vessels is approximately higher than 200; therefore we set global threshold *μ*_0_ to be 200 which means it will extract the target regions with intensity value larger than 200. By embedding the global threshold into the energy function, we define and extract the thick main artery structures of vessels better. In addition, we conceive a dynamic threshold through the role of the Gaussian kernel function, which is used to characterize the local intensity information of vessels. The local thresholds segment the tiny vessels from background more completely.

**Figure 3:**
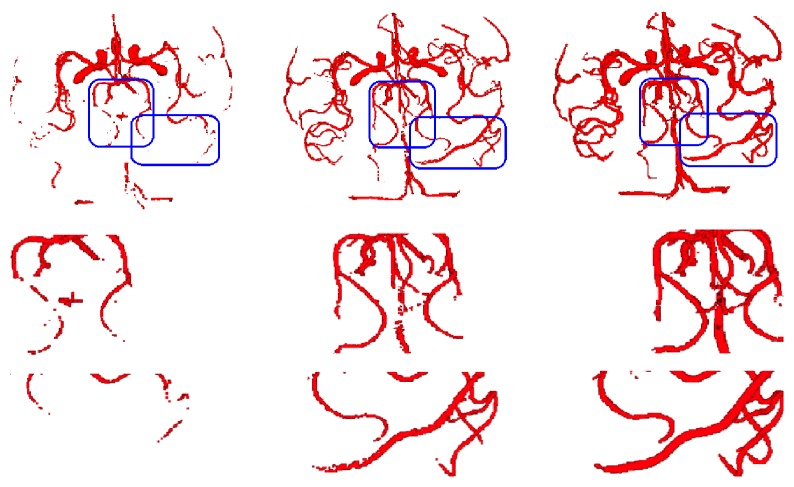
Some details. The first row represents the segmentation results by HLS, LLS, and NHLS. The second and third row are the amplified spatial details corresponding to the local region, respectively (marked with the blue boxes).

To highlight advantages of our approach, Figure 3 presents some details of Data 1. The first row is the segmentation result, and the second row and third row are the amplified spatial details corresponding to the local regions (marked with the blue boxes). The details show that the result of the HLS model loses many surrounding branches, the LLS model segments tiny branches, but branches are not continuous. The last column is the result segmented by NHLS which extracts those branches, at the same time, it is more integrated. In addition, the thick structures are hollow segmented by LLS with dynamic threshold, and our method solves the defect.

Besides the visual inspection, we also evaluate the segmentation accuracy using the Dice Similarity Coefficient (DSC), a widely used metric to evaluate segmentation algorithms for different medical image modalities [38]. Radiologists are invited to segment four sets of MRA data, and the segmentation results are as the ground truth. We, respectively, count the voxel numbers of results segmented by HLS, LLS, and our NHLS model and the voxel numbers of corresponding ground truths. The DSC is defined as

(18) $$\begin{array}{c} \text{DSC}=2\times \frac{N\left\{M\cap G\right\}}{N\left\{M\right\}+N\left\{G\right\}}\times 100, \end{array}$$

where* M* and* G* are the segmentation results and ground truths and* N* is the voxel number. And ∩ denotes the set cardinality. It has value 1 when *M* and *G* are equal and 0 when they do not share any voxel.

**Figure 4:**
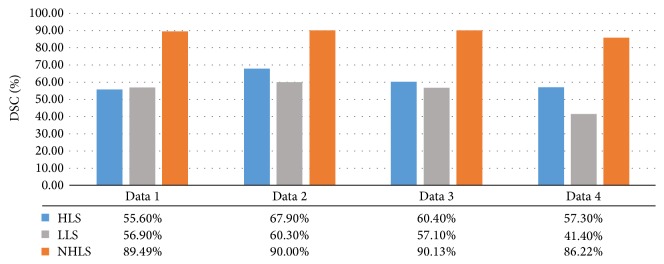
The DSC of four sets of data using three methods.

Figure 4 summarizes the average DSC for three methods. Three observations can be made from Figure 4. First, the DSC achieved by our method is over 80% for most cases. This might be because parts of the vessel were not highlighted due to the vascular disease causing disconnection among voxels in the spatial domain. Second, the average DSC of our method is 29.7%~44.8% higher than that of LLS. We think that is mainly due to not ideal segmentation of main thick vessels. Third, the average DSC of our method is 22.1%~33.9% higher than that of HLS method. We believe HLS model's poor performance is mainly due to the static intensity threshold. Although we could manually select the most suitable threshold value for evolution, it remains challenging to distinguish low contrast vessel from background.

### 4.2. Sensitivity Analysis for the Parameters

The corresponding parameters of the above experiments are *α*_1_, *α*_2_, *β*, Δ*t*, *γ*, and *ε*. Among them, three parameters *α*_1_, *α*_2_, and Δ*t* have more effects on the segmentation results. Parameters *α*_1_ and *α*_2_ are the weight coefficients of the two region terms, and they balance the roles between the global grayscale and the local information. By our test, when *α*_1_ equals *α*_2_, which means the two region terms play the same role, our segmentation results are better. The test on Data 1 is as shown in Table 1. About Δ*t*, we reference the selection of time step in [28], which considers both the speed of evolution and the error in the boundary location, and it concludes that time step Δ*t* usually is set smaller than 10. In our experiments, if the time step is bigger, the evolution can be speeded up; however, there exist more nonvessel points in the segmentation result, which affects the accuracy. Δ*t* = 2.0 is a tradeoff and suitable for this study.

## 5. Conclusions

We have introduced a new hybrid method for the automatic segmentation of cerebral vessels based on an active contour model. The joint energy terms of static and adaptive dynamic kernel within the level-set framework allow for the extraction of thick and thin vessels as well. We have evaluated our method on 10 data sets showing that approximately 80% of DSC are required, and the method performs comparably better than the other two algorithms. Our future work includes acceleration of the current method and further accuracy improvement through vascular compartment recognition.

## Figures and Tables

**Table 1:** Corresponding parameters *α*_1_, *α*_2_.

*α*_2_/*α*_1_	0.25	0.5	1	2	4
DSC (%)	82.71	85.12	89.49	84.00	80.49
